# Incidence of multiple primary cancers in a cohort of women diagnosed with breast cancer in southeast England

**DOI:** 10.1054/bjoc.2000.1603

**Published:** 2001-02

**Authors:** H S Evans, C M Lewis, D Robinson, C M J Bell, H Møller, S V Hodgson

**Affiliations:** Thames Cancer Registry, Division of Oncology, Guy's, King's and St Thomas' School of Medicine, Kings College, London, UK; Division of Medical and Molecular Genetics, Guy's, King's and St Thomas' School of Medicine, Kings College, London, UK

**Keywords:** multiple primary cancers, cancer genetics, registries, breast cancer

## Abstract

Among women in the Thames Cancer Registry database with a first breast cancer diagnosed between 1961–1995 observed numbers of subsequent cancers were compared with expected numbers and standardized incidence ratios were calculated. The occurrence of breast cancers subsequent to cancers at other sites was also examined. Women diagnosed with breast cancer before age 50 had significantly elevated risks for 9 cancer sites namely, oesophagus, stomach, lung, bone, connective tissue, breast, corpus uteri, ovary and myeloid leukaemia compared with 2 sites (corpus uteri and myeloid leukaemia) in women diagnosed at age 50 and above. Some of these associations are consistent with the effects of known inherited cancer susceptibility genes, shared environmental factors, or therapy. © 2001 Cancer Research Campaign http://www.bjcancer.com


British Journal of Cancer (2001) 84(3), 435–440
© 2001 Cancer Research Campaign
doi: 10.1054/ bjoc.2000.1603, available online at http://www.idealibrary.com on IDEAL®

http://www.bjcancer.com

### Incidence of multiple primary cancers in a cohort of women diagnosed with breast cancer in southeast England

**HS Evans[1], CM Lewis[2], D Robinson[1], CMJ Bell[1], H Møller[1] and SV Hodgson[2]**

[1]Thames Cancer Registry, Division of Oncology, Guy's, King's and St Thomas' School of Medicine, Kings College London, UK; [2]Division of Medical and Molecular Genetics, Guy's, King's and St Thomas' School of Medicine, Kings College London, UK

**Summary** Among women in the Thames Cancer Registry database with a first breast cancer diagnosed between 1961–1995 observed numbers of subsequent cancers were compared with expected numbers and standardized incidence ratios were calculated. The occurrence of breast cancers subsequent to cancers at other sites was also examined. Women diagnosed with breast cancer before age 50 had significantly elevated risks for 9 cancer sites namely, oesophagus, stomach, lung, bone, connective tissue, breast, corpus uteri, ovary and myeloid leukaemia compared with 2 sites (corpus uteri and myeloid leukaemia) in women diagnosed at age 50 and above. Some of these associations are consistent with the effects of known inherited cancer susceptibility genes, shared environmental factors, or therapy. © 2001 Cancer Research Campaign http://www.bjcancer.com

**Keywords**: multiple primary cancers; cancer genetics; registries; breast cancer

The occurrence of multiple cancers in an individual could be due to: the inheritance of rare genetic mutations which confer a strong susceptibility to the relevant cancer; inherited mutations in more common genes, conferring a less pronounced susceptibility; effects of therapy for the first cancer; misdiagnosed metastases; environmental exposure increasing the risk of both cancers; or chance. Several genes in which inherited mutations can predispose to breast cancer have been identified including *BRCA1*, *BRCA2*, *p53* and *PTEN*. In each case, mutations in the gene increase the risk of other specific cancers in addition to breast cancer. Co-occurrence of breast cancer and cancer at another site could be due to inherited mutations in genes which predispose to both cancer types. However, this may account for only a small proportion of cases of multiple cancers.

In this paper, we have used one of the world's largest population-based cancer registries to identify second cancers which occur at a higher than expected rate after the diagnosis of female breast cancer. The sequence of cancers in breast cancer patients with multiple primaries was also examined. Cases of second cancer induced by treatment or the misdiagnosis of a metastasis are clearly sequence-specific, whereas a genetic or environmental link should not greatly influence the sequence in which the cancers were diagnosed. Young age at diagnosis is a common feature of cancer in individuals with a genetic predisposition, and hence higher risks of a second cancer in younger diagnosed women than in older diagnosed women could indicate an underlying genetic susceptibility. The interval between diagnosis of the breast cancer and diagnosis of a subsequent cancer can also give an indication as to whether the association is likely to be treatment related.

*Received 11 September 2000*
*Revised 31 October 2000*
*Accepted 31 October 2000*

*Correspondence to:* H Evans

### MATERIALS AND METHODS

The Thames Cancer Registry (TCR) database was used to identify breast cancer patients with multiple primary cancers. The TCR is a population-based registry, which collects data on cancer in residents of South East England (a population of 14 million). Data collection began in 1960 in the South Thames Region and was extended in 1985 to also cover the North Thames Region. The patients registered at the TCR represent a cohort of individuals followed up from diagnosis to death, and the database currently contains over 1.5 million incident cancers. Approximately 75 000 individuals (5%), identified from the computer system at the TCR, are diagnosed with multiple tumours (excluding basal cell carcinomas of the skin). The numbers of observed second cancers can be compared with those expected using cancer rates observed in the corresponding region during the same time period.

For the analysis of multiple cancers, it is important that metastases or recurrences of the initial tumour should not be misclassified as primary tumours. The rules for accepting a second tumour as a new primary, rather than a metastasis, are well defined and nationally agreed among the UK registries. As a general rule, a new primary tumour needs to be at a different anatomical site and of a different histological type from the first tumour, or to be stated explicitly as being a new tumour by the treating clinician.

An index cohort was created by extracting all registrations of females with a first breast cancer, resident in the North or South Thames region, diagnosed between 1 January 1961 and 31 December 1995 from the TCR database. Cases were stratified by the age at diagnosis of breast cancer (under 50 years or 50 years and over) and subsequent analyses were performed separately on these two groups. The cut-off age of 50 years was chosen in order to include approximately one-quarter of cases in the younger group (22.5% of breast cancer cases were diagnosed under age 50). Within each individual, subsequent cancers were sequenced according to the date of diagnosis and the maximum number of primary cancers for any one individual was tabulated. For women diagnosed with two or more cancers subsequent to breast cancer, each cancer was analysed as an independent observation.

Non-melanoma skin cancers were excluded, because of known under-reporting (Scotto et al, 1996). Non-malignant tumours were also excluded, along with second cancers occurring within one year of the initial cancer at the same site, with the same laterality and histology. Patients with a missing date of birth were excluded, as were cancers without a year of diagnosis or information on residence of the patient at the time of diagnosis. Patients with two cancers at different sites diagnosed on the same day were not included in the analysis.

In order to identify second cancers that occur at a higher rate than expected after breast cancer, we computed person years at risk and applied appropriate population-based rates. For a given subsequent cancer site, person-years at risk were calculated from the date of diagnosis of breast cancer to the date of first diagnosis of cancer at the specified site or to the exit date (date of death, loss to follow up or 85th birthday, whichever was earlier). Patients diagnosed prior to 1 January 1971 were followed up actively, obtaining death information, until 31 December 1982. These patients were censored at this date. Patients diagnosed after this date were followed up passively through the NHS Central Registry, which provides notification of all deaths routinely to the registry. The follow up of these patients was censored at 31 December 1996. Age/period-specific cancer incidence rates for the region covered by the TCR were then applied to the cohort, to calculate the number of subsequent tumours that would be expected for each site. This number was compared with the observed number to obtain a standardized incidence ratio (SIR) estimate and 95% confidence intervals were calculated assuming a Poisson distribution. Calculations were performed using the statistical package Stata (StataCorp, 1999). A total of 40 cancer sites were studied, classified according to the 10th revision of the International Classification of Diseases (ICD) (World Health Organisation, 1992). No formal correction for multiple testing was made.

Further analyses were performed for those second tumour sites for which more than 100 cases were observed or which showed a statistically significant association with breast cancer (in either age group, with either positive or negative association). Each of these sites was taken as the index tumour, all patients with this tumour were extracted from the database and their subsequent tumours tabulated (as above) to estimate whether an excess of breast cancers was observed. For each site an age cut-off was used to classify the cases into young age of diagnosis (approximately one quarter of total cases) or older age of diagnosis.

The interval between diagnosis of the breast cancer and diagnosis of a subsequent cancer was examined to determine if an association was likely to be due to treatment such as radiotherapy. SIR estimates for subsequent cancers were calculated for intervals of 0–4 years, 5–9 years, 10–14 years and more than 15 years after the breast cancer diagnosis.

### RESULTS

A total of 145 677 women with breast cancer diagnosed between 1 January 1961 and 31 December 1995 were included in this study,

**Table 1** Standardized incidence ratios (SIR) for occurrence of subsequent cancers after initial diagnosis of cancer of the breast (expected number greater than 20 across both age groups, or statistically significant)

| | Breast cancer diagnosed at age <50 | | | Breast cancer diagnosed at age 50–84 | | |
|---|---|---|---|---|---|---|
| **Subsequent site** | ***n*** | **SIR** | **95% CI** | ***n*** | **SIR** | **95% CI** |
| Tongue | 3 | 1.08 | 0.35–3.36 | 13 | 0.75 | 0.43–1.28 |
| Mouth | 3 | 0.96 | 0.31–2.98 | 14 | 0.70 | 0.41–1.18 |
| Oesophagus | 24 | 2.39*** | 1.60–3.57 | 143 | 1.11 | 0.94–1.30 |
| Stomach | 32 | 1.83*** | 1.29–2.59 | 257 | 0.95 | 0.84–1.07 |
| Colon | 54 | 0.99 | 0.76–1.29 | 517 | 0.88** | 0.80–0.96 |
| Rectum | 27 | 0.87 | 0.60–1.27 | 263 | 0.91 | 0.80–1.02 |
| Liver | 0 | – | – | 11 | 0.39** | 0.21–0.70 |
| Gallbladder | 2 | 0.49 | 0.12–1.96 | 40 | 0.86 | 0.63–1.17 |
| Pancreas | 24 | 1.34 | 0.90–1.99 | 174 | 0.78*** | 0.67–0.90 |
| Larynx | 3 | 0.90 | 0.29–2.79 | 17 | 0.81 | 0.50–1.30 |
| Lung, bronchus | 127 | 1.49*** | 1.26–1.78 | 526 | 0.68*** | 0.62–0.74 |
| Bone | 4 | 2.79* | 1.05–7.43 | 3 | 0.46 | 0.15–1.44 |
| Connective tissue | 9 | 2.27* | 1.18–4.37 | 16 | 0.94 | 0.58–1.53 |
| Skin melanoma | 32 | 1.22 | 0.86–1.72 | 126 | 1.19 | 1.00–1.41 |
| Breast | 768 | 1.89*** | 1.76–2.03 | 1278 | 0.88*** | 0.83–0.93 |
| Cervix uteri | 31 | 0.61** | 0.43–0.87 | 103 | 0.80* | 0.66–0.97 |
| Corpus uteri | 67 | 1.29* | 1.02–1.64 | 429 | 1.68*** | 1.53–1.85 |
| Ovary | 91 | 1.29* | 1.05–1.59 | 270 | 0.89 | 0.79–1.01 |
| Bladder | 19 | 1.10 | 0.70–1.72 | 189 | 0.94 | 0.82–1.08 |
| Kidney | 10 | 0.84 | 0.45–1.56 | 80 | 0.97 | 0.78–1.21 |
| Brain/nervous system | 14 | 0.73 | 0.43–1.24 | 40 | 0.59*** | 0.43–0.80 |
| Thyroid | 12 | 1.74 | 0.99–3.07 | 25 | 0.92 | 0.62–1.36 |
| NHL | 25 | 0.96 | 0.65–1.42 | 127 | 0.77** | 0.65–0.91 |
| Multiple myeloma | 9 | 1.01 | 0.52–1.94 | 55 | 0.63*** | 0.48–0.82 |
| Lymphoid leukaemia | 3 | 0.57 | 0.18–1.77 | 48 | 0.85 | 0.64–1.13 |
| Myeloid leukaemia | 22 | 2.31*** | 1.52–3.51 | 86 | 1.39** | 1.12–1.71 |
| All sites | 1478 | 1.49*** | 1.42–1.57 | 5038 | 0.86*** | 0.84–0.88 |
| All sites excluding breast cancer | 710 | 1.21*** | 1.13–1.31 | 3760 | 0.85*** | 0.83–0.88 |

*n* = observed number of cases, * $P < 0.05$, ** $P < 0.01$, *** $P < 0.001$.

 © 2001 Cancer Research Campaign with a total of 152 453 tumours at all sites fulfilling the criteria for inclusion. In addition, 551 women had another cancer diagnosed at a different site on the same day as the breast cancer and were excluded.

### Patients with breast cancer diagnosis at age less than 50 years.

A total of 32 799 females were diagnosed with a first breast tumour under the age of 50 years during the study period. The mean follow up time was 7.5 years per woman. Multiple tumours were reported in 1448 (4.4%) cases: 1389 women had two tumours, 57 had 3 and two women had 4 tumours. 38.6% of subsequent tumours were diagnosed within 5 years of the first tumour, 25.6% within 5–9 years, 15.7% within 10–14 years and 20.2% after at least 15 years.

The numbers of subsequent tumours observed were more than expected in 18 of the 40 sites studied. Sites with a number of expected tumours greater than 20 (across all ages) or with a statistically significant result (in either age group) are shown in Table 1. The elevated risk was statistically significant ($P < 0.05$) in 9 of these sites: oesophagus (SIR 2.39, 95% CI 1.60–3.57), stomach (SIR 1.83, 95% CI 1.29–2.59), lung (SIR 1.49, 95% CI 1.26–1.78), bone (SIR 2.79, 95% CI 1.05–7.43), connective tissue (SIR 2.27, 95% CI 1.18–4.37), breast (SIR 1.89, 95% CI 1.76–2.03), corpus uteri (SIR 1.29, 95% CI 1.02–1.64), ovary (SIR 1.29, 95% CI 1.05–1.59) and myeloid leukaemia (SIR 2.31, 95% CI 1.52–3.51). The increased risk for a thyroid tumour was of borderline significance (SIR 1.74, 95% CI 0.99–3.07, $P = 0.052$). A significantly reduced risk was seen only for cervix uteri (SIR 0.61, 95% CI 0.43–0.87). The overall SIR for developing a subsequent non-breast cancer after diagnosis of breast cancer under 50 years of age was 1.21 (95% CI 1.13–1.31).

The SIR estimates by interval from first diagnosis are shown in Figure 1, for sites with more than 10 observed tumours and with a significant association. Cancer of the oesophagus showed a marked increase at 15 years or more after the breast cancer diagnosis. The increase in myeloid leukaemia was restricted to the first 5 years after the breast cancer diagnosis. Risks for other cancer sites were relatively constant, regardless of the time elapsed since the initial cancer.

### Patients with breast cancer diagnosis at age 50 years and above

A total of 112 878 females were diagnosed with a first breast tumour between age 50 and 84 years (77.5% of the total). The mean follow up time was 5.2 years per person, substantially lower than the 7.5 years of follow-up for young onset cases. Multiple tumours were reported in 5034 (4.5%) cases: 4815 women had 2 tumours, 205 had 3 and 14 had 4. 53.8% of subsequent tumours were diagnosed within 5 years of the first cancer, 27.4% within 5–9 years, 11.3% within 10–14 years and 7.5% after at least 15 years.

Of the 40 subsequent tumour sites observed, 8 showed an excess of observed cancers. Two sites with an expected number of tumours greater than 20 showed a significant excess: corpus uteri (SIR 1.68, 95% CI 1.53–1.85) and myeloid leukaemia (SIR 1.39, 95% CI 1.12–1.71). The increase in corpus uteri cancers was seen in all 4 time intervals after the breast cancer diagnosis, whereas the rise in myeloid leukaemia was restricted to the first 5 years. Significantly reduced SIRs were found at 9 sites: colon, liver, pancreas, lung, breast, cervix uteri, brain, NHL (non- Hodgkin's lymphoma) and multiple myeloma.

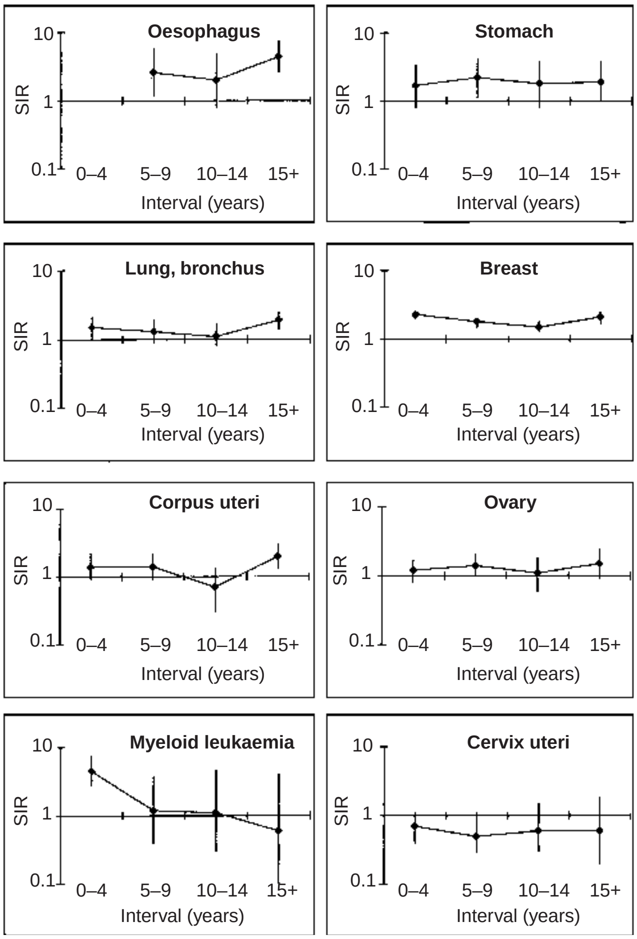

**Figure 1** Standardized incidence ratios (SIR) for cancers following breast cancer diagnosed before the age of 50, by interval since diagnosis

**Table 2** Standardized incidence ratios (SIR) for occurrence of breast cancer after diagnosis of initial cancer at a young age, for sites with more than 10 subsequent breast cancers or showing a statistically significant association

| | Age cut-off (years) | Observed number of cases | SIR | 95% CI |
|---|---|---|---|---|
| Oesophagus | 65 | 12 | 1.36 | 0.77–2.40 |
| Stomach | 65 | 22 | 1.24 | 0.82–1.89 |
| Colon | 65 | 150 | 1.18* | 1.04–1.43 |
| Rectum | 60 | 58 | 1.18 | 0.91–1.52 |
| Lung, bronchus | 60 | 54 | 1.60*** | 1.23–2.09 |
| Bone | 20 | 1 | 10.48** | 1.48–74.38 |
| Skin melanoma | 40 | 18 | 1.12 | 0.70–1.77 |
| Cervix uteri | 40 | 28 | 0.90 | 0.62–1.30 |
| Corpus uteri | 55 | 84 | 0.84 | 0.68–1.05 |
| Ovary | 55 | 77 | 1.12 | 0.90–1.40 |
| Bladder | 65 | 73 | 1.08 | 0.86–1.35 |
| Thyroid | 40 | 13 | 1.97* | 1.14–3.39 |
| NHL | 55 | 18 | 0.64 | 0.40–1.02 |
| Multiple myeloma | 65 | 1 | 0.09* | 0.01–0.61 |

* $P < 0.05$, ** $P < 0.01$, *** $P < 0.001$.

### Breast cancer as a subsequent tumour

There was a significant increase in breast cancer after a number of cancers that were diagnosed at a young age (Table 2): colon (SIR 1.18, 95% CI 1.04–1.43), lung (SIR 1.60, 95% CI 1.23–2.09), bone (SIR 10.5, 95% CI 1.48–74.4) and thyroid (SIR 1.97, 95% CI

© 2001 Cancer Research Campaign

1.14–3.39). Significantly fewer breast cancers than expected were observed after a young diagnosis of multiple myeloma (SIR 0.09, 95% CI 0.01–0.61).

### DISCUSSION

Comparing women with breast cancer diagnosed under age 50 and over age 50, we note an increased risk of second cancer occurring in the younger group. It is difficult to distinguish between cases of multiple cancers due to a genetic susceptibility and those due to common environmental factors. However, the majority of associations were seen with breast cancer diagnosed under age 50, and early age at onset is considered a hallmark of inherited cancers (Schottenfeld, 1996).

It is possible that the overall significant decrease in tumours reported after an older diagnosis of breast cancer may be due to under-ascertainment. It is conceivable that medical surveillance is reduced in older patients. Older women may not have such aggressive treatment as the younger cases and so might develop fewer treatment-related cancers.

The common sites of metastasis from breast cancer are bone, lung, liver and brain. Lung and bone were both significantly over-represented in the younger age group and it is possible that some of these were misclassified as primary tumours, but none of these sites were increased in the older group.

We would expect tumours induced by therapy for breast cancer to be over-represented in both groups. Solid tumours resulting from radiotherapy include cancers of the lung, bone, oesophagus, thyroid, salivary gland and connective tissue near the irradiation site. These usually occur 10 or more years after radiation, whereas leukaemia typically occurs within 5 years of radiotherapy (Boice et al, 1996). Secondary myeloid leukaemia has also been described after chemotherapy for breast cancer (Kollmannsberger et al, 1998).

Myeloid leukaemia was significantly increased within five years of diagnosis of breast cancer (Figure 1). When the tumour sequence was reversed, breast cancer was not seen in excess after diagnosis of myeloid leukaemia. The associations with cancer of the oesophagus and connective tissue were not seen in the reverse order, indicating that they may also be due to treatment for breast cancer. There is evidence that tamoxifen treatment may increase the risk of cancer of the corpus uteri in some women (Andersson et al, 1991; Gail et al, 1999), which may account for some of the excess seen in this study.

Inherited mutations in genes predisposing for breast cancer (*BRCA1*, *BRCA2*, *PTEN*, *p53*) increase risks of other cancers also and will therefore contribute to the relative risks of subsequent cancers, particularly for breast cancer diagnosed at a young age (Ford et al, 1994; Eeles, 1995; Breast Cancer Linkage Consortium, 1999; De Vivo et al, 2000). The penetrance for ovarian cancer in

**Table 3** Summary of relative risks found in other cancer registry studies of multiple cancer following diagnosis of breast cancer

| **Area** | **South East England** | **Denmark** | **Connecticut, USA** | **Finland** | **Slovenia** |
|---|---|---|---|---|---|
| Reference | Current study | Ewertz and Mouridsen (1985) | Harvey and Brinton (1985) | Teppo et al (1985) | Volk and Pompe-Kirn (1997) |
| Period | 1961–1995 | 1943–1980 | 1935–1982 | 1953–1979 | 1961–1985 |
| No. of breast cancer cases | 32 799 | 54 964 | 41 109 | 26 617 | 8917 |
| Age cut-off | Under 50y | All cases | Under 45y | All cases | All cases |
| Subsequent sites (SIR): | | | | | |
| Tongue | 1.08 | 0.7 | 1.8 | NR | NR |
| Mouth | 0.96 | 1.1 | 2.2 | NR | 0.7 |
| Oesophagus | 2.39*** | 1.0 | 1.3 | 0.78 | 1.2 |
| Stomach | 1.83*** | 1.0 | 1.1 | 1.11 | 0.9 |
| Colon | 0.99 | 1.1 | 1.6* | 1.36* | 1.1 |
| Rectum | 0.87 | 1.0 | 1.9* | 0.97 | 1.2 |
| Liver | – | 0.7* | 1.5 | NR | 0.7 |
| Gallbladder | 0.49 | NR | NR | 0.43* | 0.9 |
| Pancreas | 1.34 | 1.0 | 1.8 | NR | 0.6 |
| Larynx | 0.90 | 1.0 | 0.7 | NR | 3.0 |
| Lung, bronchus | 1.49*** | 1.4* | 1.7* | 1.67** | 1.6* |
| Bone | 2.79* | 2.3* | 2.6 | NR | 1.2 |
| Connective tissue | 2.27* | 2.1* | 1.5 | NR | 2.0 |
| Skin melanoma | 1.22 | 1.4* | 1.8 | NR | 2.7* |
| Breast | 1.89*** | NR | 5.4* | NR | 1.4* |
| Cervix uteri | 0.61** | 0.9 | 1.3 | 0.95 | 1.1 |
| Corpus uteri | 1.29* | 1.0 | 1.1 | 1.33* | 1.6* |
| Ovary | 1.29* | 1.3* | 2.6* | 1.73*** | 2.3* |
| Bladder | 1.10 | 0.9 | 0.6 | 1.65* | 1.1 |
| Kidney | 0.84 | 1.2 | 0.5 | NR | 1.0 |
| Brain/nervous system | 0.73 | 0.8 | 1.0 | NR | NR |
| Thyroid | 1.74 | 1.1 | 3.2* | 1.95* | 2.5* |
| NHL | 0.96 | 1.0 | 1.1 | NR | 0.9 |
| Multiple myeloma | 1.01 | 0.7 | 1.4 | NR | 0.9 |
| Lymphoid leukaemia | 0.57 | 1.0[1] | 0.0[1] | NR | NR |
| Myeloid leukaemia | 2.31*** | 2.5[2*] | 2.1[2] | NR | NR |

NR = not reported * $P$<0.05, ** $P$<0.01, *** $P$<0.001. 1 = chronic lymphocytic leukaemia, 2 = acute nonlymphocytic leukaemia. Denmark, Connecticut and Slovenia studies stated only $P$ < 0.05.

© 2001 Cancer Research Campaign

*BRCA1* mutation carriers is approximately 0.6 (Easton et al, 1995), compared to the lifetime population risk of 0.013 (RR = 46.15). If we assume that 3% of breast cancer cases diagnosed under age 50 are *BRCA1* mutation carriers (Peto et al, 1999) and that the remaining cases have the population risk for ovarian cancer, then the predicted SIR for ovarian cancer subsequent to breast cancer would be 2.35, which is higher than our estimate of 1.29 (95% CI 1.05–1.59). These calculations are approximations, and ignore the effect of ovarian cancer in *BRCA2* mutation carriers, or possibly lower survival rates for breast cancer cases, but they suggest that mutations in *BRCA1* and *BRCA2* could fully account for the observed increased risk of ovarian cancer following breast cancer.

In our study, thyroid cancer risks are increased after breast cancer diagnosed under age 50 (SIR 1.74). However, the increase is unlikely to be due to radiotherapy for breast cancer, since the risk of breast cancer is also significantly increased after thyroid cancer diagnosed under age 40 (SIR 1.97). This supports findings reported previously (Ron et al, 1984; Vassilopoulou-Sellin et al, 1999). Genetic syndromes associated with a predisposition to thyroid cancer include Cowden disease (Liaw et al, 1997); ataxia telangiectasia (Swift et al, 1987); inherited multiple endocrine neoplasia type II (Schimke, 1984); familial adenomatous polyposis (FAP) and hereditary non-polyposis colorectal cancer (HNPCC) (Houlston and Stratton, 1995). However, few of these are considered to be an important cause of breast cancer (Warren et al, 1992; Fitzgerald et al, 1997; Freihoff et al, 1999; Inskip et al, 1999).

Stomach cancer is associated with *BRCA2* mutations as well as a number of other genetic syndromes (FAP, HNPCC, Peutz-Jegher's syndrome and Cowden disease), but these are rare and it is unlikely that they can explain the excess risk associated with breast cancer (Bevan and Houlston, 1999). A Swedish study (Adami et al, 1984) also reported an excess risk of stomach cancer, which was attributed to the fact that a high proportion of stomach cancer was diagnosed incidentally at autopsy.

Some associations found will be due to shared environmental predisposing factors. Cancers of the breast and the corpus uteri share common reproductive risk factors such as nulliparity, early menarche and late onset of menopause. The lack of an increased risk of breast cancer after cancer of the corpus uteri could be explained by the fact that treatment for cancer of the corpus uteri may include removal of the ovaries, which would decrease the subsequent breast cancer risk (Henderson et al, 1996). This is also true for breast cancer occurring after ovarian cancer. Similarly, the risk of a second breast cancer reported in this study is likely to be lower than the true risk as it does not account for the fact that a proportion of women will have had mastectomies. There were significantly fewer than expected cases of cervical cancer after breast cancer, and of breast cancer after cervical cancer. A small deficit of cervical cancer cases following breast cancer was seen in a study from the Birmingham Regional Cancer Registry (Prior and Waterhouse, 1981), and Peto et al (1996) found decreased mortality rates from cervical cancer in first degree relatives of breast cancer cases.

The increased risk for lung cancer after breast cancer observed in this study has been reported by other cancer registry studies. However, the reasons for the association have not been fully explained. Smoking is not thought to be a risk factor for breast cancer (Palmer and Rosenberg, 1993). Radiotherapy may account for some of the association, although a Connecticut study (Harvey and Brinton, 1985) reported an increase in women who did not have radiotherapy as well as in those that did.

Other cancer registries have reported similar studies of women with breast cancer, four of which are summarized in Table 3. At least 3 of the 4 report increased risks for cancers of the lung, corpus uteri, ovary and thyroid, as reported in our study.

We have identified several sites for which women diagnosed with breast cancer are at higher risk of developing cancer. Associations with some sites could be due to known genes (ovary), shared environmental factors (corpus uteri), or the effects of therapy (myeloid leukaemia). Other associations, such as thyroid and stomach, are more difficult to explain. They may be artefactual, or they could be caused by more common but less penetrant genetic mutations causing only a moderately raised susceptibility to these cancers.

### ACKNOWLEDGEMENTS

The authors would like to thank Dr Rosalind Eeles for her valuable comments. This study was supported by grants from the Special Trustees of Guy's Hospital and the Dunhill Medical Trust.

### REFERENCES

Adami HO, Bergkvist L, Krusemo U and Persson I (1984) Breast cancer as a risk factor for other primary malignant diseases. A nationwide cohort study. *J Natl Cancer Inst* **73**: 1049–1055

Andersson M, Storm HH and Mouridsen T (1991) Incidence of new primary cancers after adjuvant tamoxifen therapy and radiotherapy for early breast cancer. *J Natl Cancer Inst* **83**: 1013–1017

Bevan S and Houlston RS (1999) Genetic predisposition to gastric cancers. *QJM* **92**: 5–10

Boice JD, Land CE and Preston DL (1996) Ionizing radiation. In: *Cancer Epidemiology and Prevention*. 2nd ed, Schottenfeld D, Fraumeni JF (ed), pp 319–354. Oxford University Press: New York

Breast Cancer Linkage Consortium (1999) Cancer Risks in BRCA2 mutation carriers. *J Natl Cancer Inst* **91**: 1310–1316

De Vivo I, Gertig DM, Nagase S, Hankinson SE, O'Brien R, Speizer FE, Parsons R and Hunter DJ (2000) Novel germline mutations in the PTEN tumour suppressor found in women with multiple cancers. *J Med Genet* **37**: 336–341

Easton DF, Ford D and Bishop DT (1995) Breast and ovarian cancer incidence in BRCA1 mutation carriers. Breast Cancer Linkage Consortium. *Am J Hum Genet* **56**: 265–271

Eeles RA (1995) Germline mutations in the TP53 gene. *Cancer Survey* **25**: 101–124

Ewertz M and Mouridsen HT (1985) Second cancer following cancer of the female breast in Denmark, 1943–80. *Natl Cancer Inst Monogr* **68**: 325–329

Fitzgerald MG, Bean JM, Hegde SR, Unsal H, MacDonald DJ, Harkin DP, Finkelstein DM, Isselbacher KJ and Haber DA (1997) Heterozygous ATM mutations do not contribute to early onset breast cancer. *Nat Genet* **15**: 307–310

Ford D, Easton DF, Bishop DT, Narod SA and Goldgar DE (1994) Risks of cancer in BRCA1 mutation carriers: Breast Cancer Linkage Consortium. *Lancet* **343**: 692–695

Freihoff D, Kempe A, Beste B, Wappenschmidt B, Kreyer E, Hayashi Y, Meindl A, Krebs D, Wiestler OD, von Deimling A and Schmutzler RK (1999) Exclusion of a major role for the PTEN tumour suppressor gene in breast carcinomas. *Br J Cancer* **79**: 754–758

Gail MH, Costantino JP, Bryant J, Croyle R, Freedman L, Helzlsouer K and Vogel V (1999) Weighing the risks and benefits of tamoxifen treatment for preventing breast cancer. *J Natl Cancer Inst* **91**: 1829–1846

Harvey EB and Brinton LA (1985) Second cancer following cancer of the breast in Connecticut, 1935–1982. *Natl Cancer Inst Monogr* **68**: 99–109

Henderson BE, Pike MC, Bernstein L and Ross RK (1996) Breast cancer. In: *Cancer Epidemiology and Prevention*. 2nd ed, Schottenfeld D, Fraumeni JF (ed), pp 1022–1039. Oxford University Press: New York

Houlston RS and Stratton MR (1995) Genetics of non-medullary thyroid cancer. *QJM* **88**: 685–693

Inskip HM, Kinlen LJ, Taylor AM, Woods CG and Arlett CF (1999) Risk of breast cancer and other cancers in heterozygotes for ataxia-telangiectasia. *Br J Cancer* **79**: 1304–1307

© 2001 Cancer Research Campaign 

Kollmannsberger C, Hartmann JT, Kanz L and Bokemeyer C (1998) Risk of secondary myeloid leukaemia and myelodysplastic syndrome following standard-dose chemotherapy or high-dose chemotherapy with stem cell support in patients with potentially curable malignancies. *J Cancer Res Clin Oncol* **124**: 207–214

Liaw D, Marsh DJ, Li J, Dahia PL, Wang SI, Zheng Z, Bose S, Call KM, Tsou HC, Peacocke M, Eng C and Parsons R (1997) Germline mutations of the PTEN gene in Cowden disease, an inherited breast and thyroid cancer syndrome. *Nat Genet* **16**: 64–67

Palmer JR and Rosenberg L (1993) Cigarette smoking and the risk of breast cancer. *Epidemiol Rev* **15**: 145–156

Peto J, Easton DF, Matthews FE, Ford D and Swerdlow AJ (1996) Cancer mortality in relatives of women with breast cancer: the OPCS study. *Int J Cancer* **65**: 275–283

Peto J, Collins N, Barfoot R, Seal S, Warren W, Rahman N, Easton DF, Evans C, Deacon J and Stratton MR (1999) Prevalence of BRCA1 and BRCA2 gene mutations in patients with early-onset breast cancer. *J Natl Cancer Inst* **91**: 943–949

Prior P and Waterhouse JA (1981) Multiple primary cancers of the breast and ovary. *Br J Cancer* **44**: 628–636

Ron E, Curtis R, Hoffman DA and Flannery JT (1984) Multiple primary breast and thyroid cancer. *Br J Cancer* **49**: 87–92

Schimke RN (1984) Genetic aspects of multiple endocrine neoplasia. *Ann Rev Med* **35**: 25–31

Schottenfeld D (1996) Multiple primary cancer. In: *Cancer Epidemiology and Prevention*. 2nd ed, Schottenfeld D and Fraumeni JF (eds), pp 1370–1387. Oxford University Press: New York

Scotto J, Fears TR, Kraemer KH and Fraumeni JF (1996) Nonmelanoma skin cancer. In: *Cancer Epidemiology and Prevention*. 2nd ed, Schottenfeld D and Fraumeni JF (eds) pp 319–354. Oxford University Press: New York

StataCorp (1999) *Stata Statistical Software: Release 6.0*. Stata Corporation: College Station

Swift M, Reitnauer PJ, Morrell D and Chase CL (1987) Breast and other cancers in families with ataxia-telangiectasia. *N Engl J Med* **316**: 1289–1294

Teppo L, Pukkala E and Saxen E (1985) Multiple cancer – an epidemiological exercise in Finland. *J Natl Cancer Inst* **75**: 207–217

Vassilopoulou-Sellin R, Palmer L, Taylor S and Cooksley CS (1999) Incidence of breast carcinoma in women with thyroid carcinoma. *Cancer* **85**: 696–705

Volk N and Pompe-Kirn V (1997) Second primary cancers in breast cancer patients in Slovenia. *Cancer Causes and Control* **8**: 764–770

Warren W, Eeles RA, Ponder BA, Easton DF, Averill D, Ponder MA, Anderson K, Evans AM, DeMars R and Love R (1992) No evidence of germline mutations in exons 5–9 of the p53 gene in 25 breast cancer families. *Oncogene* **7**: 1043–1046

World Health Organisation (1992) *International Statistical Classification of Diseases and Related Health Problems, Tenth Revision*. WHO: Geneva.

 *© 2001 Cancer Research Campaign*
